# Developing and Establishing Biomechanical Variables as Risk Biomarkers for Preventable Gait-Related Falls and Assessment of Intervention Effectiveness

**DOI:** 10.3389/fspor.2021.722363

**Published:** 2021-09-22

**Authors:** Mark D. Grabiner, K.R. Kaufman

**Affiliations:** ^1^Biomechanics and Clinical Biomechanics Laboratory, Department of Kinesiology and Nutrition, University of Illinois at Chicago, Chicago, IL, United States; ^2^Motion Analysis Laboratory, Department of Orthopedic Surgery, Mayo Clinic, Rochester, MN, United States

**Keywords:** injury, intervention, perturbation training, prevention, risk factors

## Abstract

The purpose of this review is to position the emerging clinical promise of validating and implementing biomechanical biomarkers of falls in fall prevention interventions. The review is framed in the desirability of blunting the effects of the rapidly growing population of older adults with regard to the number of falls, their related injuries, and health care costs. We propose that biomechanical risk biomarkers may be derived from systematic study of the responses to treadmill-delivered perturbations to both identify individuals with a risk of specific types of falls, such as trips and slips as well as quantifying the effectiveness of interventions designed to reduce that risk. The review follows the evidence derived using a specific public health approach and the published biomedical literature that supports trunk kinematics as a biomarker as having met many of the criteria for a biomarker for trip-specific falls. Whereas, the efficacy of perturbation training to reduce slip-related falls by older adults appears to have been confirmed, its effectiveness presently remains an open and important question. There is a dearth of data related to the efficacy and effectiveness of perturbation training to reduce falls to the side falls by older adults. At present, efforts to characterize the extent to which perturbation training can reduce falls and translate the approaches to the clinic represents an important research opportunity.

## Introduction

Over 60 years ago, although falls and fall-related injuries among older adults were concerning issues, the published biomedical literature addressing the issues was “very meager” (Sheldon, 1960). This is easily confirmed. A PubMed search (25 May 2021) using the search terms “(falls OR falling) AND (older adults OR elderly) AND (prevention OR intervention)” returned 29,705 titles since 1956. Since 1956 the number of papers published annually has doubled approximately every 6.5 ± 2.7 years. So, the biomedical literature addressing issues related to falls and fall-related injuries among older adults is no longer meager.

Recently, the question was asked “Are we moving the needle on fall prevention?” (Hicks, 2019). The answer, according to the author was a qualified yes; the motion of the needle was said to be in a positive direction. It seems, however, that there is a growing gap between (1) the rate at which the number of older adults, who, by virtue of their age and the relentless effects of biological aging, are at increased risk of falls and injuries, is increasing and (2) the rate at which the needle may be moving and the extent to which it is moving with regard to fall prevention.

We agree that the overall motion of the needle has been in the positive direction. However, the magnitude of its motion is challenging to assess. The full range of possible motion for the needle and its current position within the range have not been, perhaps cannot be, estimated. Nevertheless, referencing Tinetti et al. (1988), Sherrington et al. (2019) wrote that “at least one-third of community dwelling people over age 65 years of age fall each year.” In their paper, Tinetti et al. (1988) referenced Prudham and Evans (1981) and Campbell et al. (1981). Thus, based on the persistence of the “one-third” statistic, as general as it may be, one may legitimately wonder about the actual extent to which the needle has been moved in over four decades.

The increase in the population of community dwelling people 65 years of age and older since the early 1980s translates to a large increase in the estimated absolute number of falls. Furthermore, as will be addressed in another section of this paper, the absolute number of falls by older adults, particularly injurious falls, are reasonably expected to increase with the growth of the population of older adults who at risk of falling as well as the number of them who do fall.

It seems reasonable to ask if the effectiveness of fall prevention intervention has reached or is approaching its maximum. The set of 355 US national health and well-being objectives for the coming decade has explicit objectives to both decrease emergency room visits due to falls by older adults and to decrease fall-related deaths by older adults (Healthy People, 2030). This may reflect that there are those who believe there remains room for improvement. Therefore, the question related to whether the effectiveness of fall prevention intervention has reached or is approaching its maximum is quite practical, the long-term impact of which ranges from the personal to the societal levels. The rate at which the older adult population is presently growing will, expectedly, increase the number of falls by older adults that occur annually. This, in turn, will increase the expected number of injurious falls. Again, in turn, this will increase the health- and economic-/financial-related impact of these falls.

The purpose of this review is to address these issues from the point of view that it is both desirable and achievable to slow, to the greatest extent possible, the effects of an already ongoing exponential growth of the population of older adults with regard to falls, particularly injurious falls, and their associated health-care costs. We propose that an effective means by which this may be approached is by greater specificity in fall-prevention interventions. That is, considering the stepping responses that may contribute to avoiding a fall following slips, trips, and falls to the side, as being separate motor skills that may be acquired, or learned, through practice. We will summarize the approach that we have implemented to identify biomechanical variables that are causally related to trip-related falls, clinically-modifiable, and that, following modification, convincingly appear to possess efficacy and effectiveness with regard to decreasing prospectively measured trip related falls by older adults. Collectively, a set of biomechanical variables has emerged that may qualify as risk biomarkers for trip-related falls. This approach is generalizable to other types of falls such as slips and falls to the side, which along with trip-related falls, share a common feature. The common feature is that in many cases, a fall may be prevented by a temporally and spatially appropriate stepping response.

When we set out to answer questions about the biomechanical causes of gait-related falls by older adults it was with the long-term goal of reducing the incidence of these falls, specifically trip-related falls. A key motivation was the belief that once a trip had occurred, the trip-related fall, or at least some of them, could be prevented by performing a spatially and temporally appropriate compensatory strategy, specifically a stepping response. We reasoned that identifying biomechanical variables that could be shown to be causally associated with failed stepping responses and, importantly, were clinically modifiable, could be followed by determining the extent to which clinically modifying these variables would decrease the prospectively measured incidence of a trip-related falls by older adults. Decreasing the incidence of trip-related falls by older adults would consequently lead to fewer trip-related injuries.

**Figure 1:**
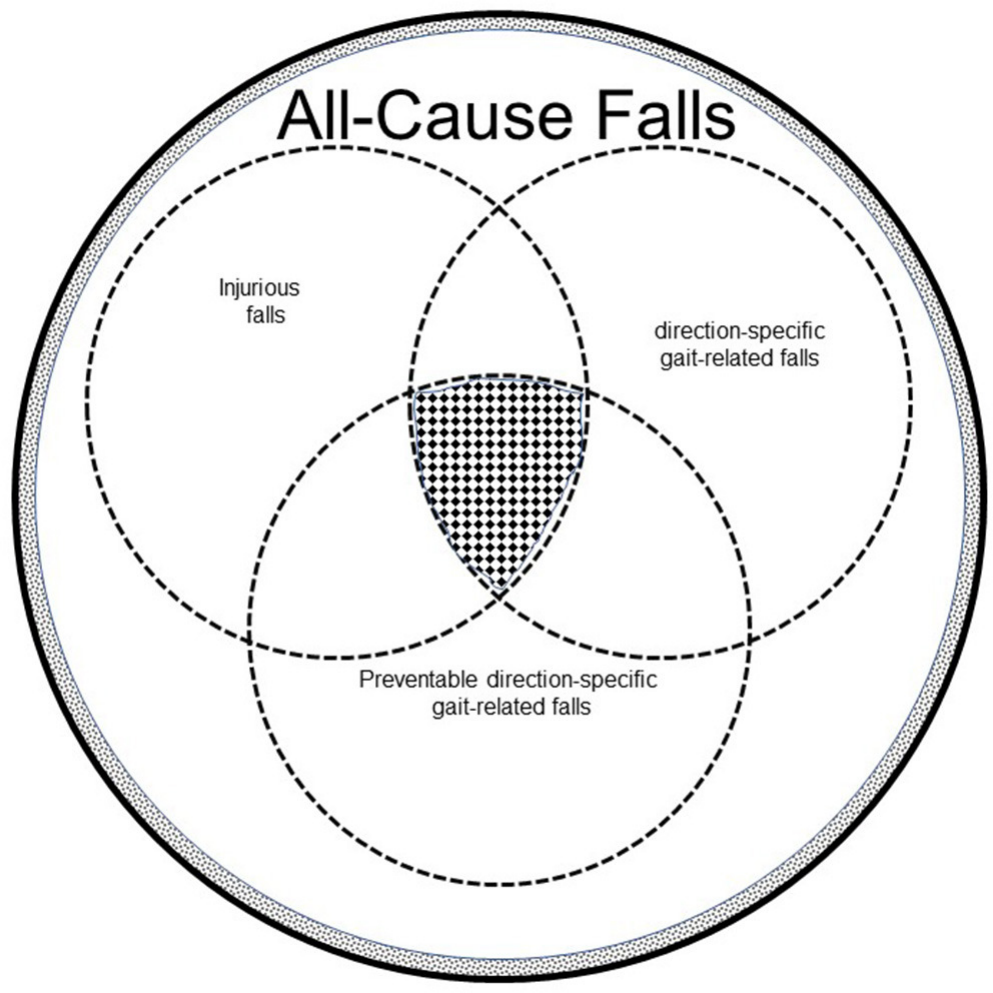
The total encircled area represents all-cause falls by all older adults. The white area represents all-cause falls by independent community dwelling older adults, who represent 95% of older adults in the United States (Administration on Aging, 2012, http://www.aoa.gov/Aging_Statistics/Profile/2012/docs/2012profile.pdf). The dotted area represents all-cause falls by older adults living in assisted living facilities. Direction-specific gait-related falls are a subset of all-cause falls. Slips and trips, direction-specific falls, that occur both in and outside the home, may generally account for about 50% gait-related falls by independent community-dwelling older adults (Berg et al., 1997; Crenshaw et al., 2017). A substantial number of falls by independent community-dwelling older adults are laterally-directed (Crenshaw et al., 2017). Because laterally-directed falls may have recovery solutions that involve stepping responses, the percentage of preventable direction-specific gait-related falls is increased. Of these gait-related falls, some proportion leads to injury, both minor and serious, the latter of which can be life-threatening. Of these direction-specific gait-related falls by independent community-dwelling older adults, some are demonstrably preventable through various clinical interventions. The hatched area at the intersection of injurious falls, direction-specific gait-related falls. and preventable, direction-specific gait-related falls represents out target for perturbation-based intervention.

We first described the biomechanical differences between successful and failed recoveries by older adults following a laboratory-induced trip (Pavol et al., 2001). In a separate paper, we described an experimental protocol in which a treadmill was used to deliver a perturbation to a standing subject and that required a forward-directed step to avoid falling (Owings et al., 2001). Failed recovery efforts by older adults following these perturbations shared general biomechanical mechanisms of failed recoveries following a laboratory-induced trip during overground walking (Pavol et al., 2001). Although this perturbation did not induce a trip as seen during overground walking by obstructing the forward progression of the swing leg, it seemed to be a reasonable, and practical model for a protocol that is not clinically-friendly. A seminal experimental result was that exposure to a single, treadmill-delivered, trip-specific perturbation was associated with changes to key biomechanical variables that led to a successful recovery for 75% of women following subsequent within-session trip-specific perturbations (Owings et al., 2001). These trip-specific perturbations were delivered as participants stood on the treadmill belt and, thus, do not include an actual obstruction of the forward motion of the swing limb as would be associated with a tripping event. Rather, it is the biomechanics of the initial recovery step, the trunk and of the weight-acceptance phase of the recovery that are “trip-specific.” This finding was consistent with predictions in accordance with the specificity of training principle (Henry, 1968). This principle holds, that “motor skill performance during a ‘test' condition is enhanced when both the sensorimotor and environmental contexts of the ‘practice' and test conditions are similar” (Grabiner et al., 2014). Collectively, our early hypotheses and results contributed to a conceptual representation of our long-term goal of reducing the incidence of trip-related falls (Figure 1).

Overall, the experimental approach that informed our subsequent approach to fall prevention intervention was that described in a paper titled “Public Health Model of a Scientific Approach to Prevention” (Mercy et al., 1993). Notably, although this approach was presented in a context related to violence prevention, the very same model was presented 17 years later in a paper titled “An older adults research agenda from a public health perspective” (Stevens et al., 2010). The basis of the model was a four element pathway, the first of which is comprehensive description of the problem(s) and its/their implication(s), including both of the problem being successfully or not successfully addressed. The second element involves the identification of risk factors and causes, particularly causes that are modifiable. The third element is the implementation of the results from the second element to design interventions, target interventions, and evaluate the efficacy and effectiveness of the interventions. Here, efficacy reflects how well an intervention works under “ideal and controlled circumstances” such as those found under laboratory conditions. In contrast, effectiveness reflects how well the intervention works in the more messy “real world” (Singal et al., 2014). Lastly, the demonstrably effective intervention and implemented. These are topics to which we shall return.

In particular, we advocate here a transition toward the clinical inclusion of valid biomechanical biomarkers for falls with validated risk factors for falls. Generally, a risk factor is a characteristic or an exposure that increases the probability of a negative outcome. On the other hand, a biomarker is an objective and quantitative measure of biological structure, function, and/or process, the purpose of which is to evaluate health status and/or response(s) to intervention(s) (Micheel and Ball, 2010). There is a distinction, and potentially meaningful difference between risk factors and biomarkers. By definition, although biomarkers may be shown to be risk factors, risk factors are not necessarily biomarkers.

Fall-risk assessment has historically consisted of assessing the presence of risk factors. Thus, in the present context, a risk factor may be considered an attribute, characteristic, or exposure that increases the probability of a fall. Common fall risk factors include muscle weakness, gait deficits, and balance deficits (Rubenstein and Josephson, 2002). Each of these risk factors can be, and are, measured in numerous ways. In addition, each may be, clinically modifiable via exercise and, in some cases have been shown to be associated with falls, fall rate, and fall-related injury. Other risk factors that are associated with of falls but that are not modifiable include one's sex, age, and history of previous falls.

## Biomarkers for falls

There has been an increase in the frequency with which the terms “fall” and “biomarker” have been conjoined in the published literature. Gait-related biomechanical variables have been suggested as potential biomarkers for Parkinson's disease (Horak and Mancini, 2013), dementia (Montero-Odasso et al., 2017), cognitive impairment (Beauchet et al., 2013), Alzheimer's Disease (Varma et al., 2021) osteoarthritis (Mezghani et al., 2017), and ALS (Inam et al., 2010). Further, the term biomarker has been used in the context of Parkinson's disease-related falls; (Brodie et al., 2014). Interestingly, serum levels of malondealdehyde, a biomarker for oxidative stress, was reported to be prospectively associated with falls (Verghese and Ayers, 2017). Based on this result the authors recommended malandealdehyde for further study, “as a fall *risk biomarker*” (italics added for emphasis) and as “a potential target to prevent falls.” The term risk biomarker has a very specific connotation.

There are eight recognized categories of biomarkers, the uses of which include, for example, diagnosis, prognosis, exposure, surveillance, and risk/susceptibility (FDA-NIH Biomarker Working Group, 2016). Risk biomarkers, aka susceptibility biomarkers, indicate the potential for developing a disease or medical condition in an individual for whom clinically apparent disease or medical condition is absent (FDA-NIH Biomarker Working Group, 2016). Examples given are women who inherit the BRCA1 and BRCA2 genes, and who are at significantly higher lifetime risk of breast and ovarian cancer, and the APOEe4 gene that increases the risk of Alzheimer's disease. In these examples of risk biomarkers, a specific gene is attributed as the cause for the clinical outcome and the associated disease risk present from the time of fertilization, decades before clinical manifestations of the diseases.

General requirements for clinical biomarkers include their capacity to be accurately measured (analytical validity), to accurately estimate structural/functional status (clinical validity), having an acceptable risk-benefit ratio (clinical utility), being clinically feasible (i.e., being time-effective and cost-effective), and being non-invasive (Ferlini et al., 2013). With respect to biomechanical biomarkers for falls, we propose three additional “ideal” criteria. First, a biomechanical biomarker for falls should ideally be established as causally related to falls. This criterion is based on the notion that the clinical benefit of monitoring a biomarker that bears no, or little influence on the outcome, in this case falls, may have a high cost:benefit ratio. Secondly, a biomechanical biomarker for falls should ideally be clinically modifiable. This criterion is based on the notion that the clinical benefit of monitoring a biomarker that is not sensitive to some type of intervention, will not influence the outcome, i.e., falls, and consequently have a high cost:benefit ratio. Thus, following a targeted intervention, a biomarker established as causally related to falls should be altered and this change in the biomarker should be accompanied by an appropriate directional change in fall risk. Thirdly, a biomechanical biomarker for falls should ideally demonstrate direction specificity. That is, the biomarker should reflect the risk for a specific type of fall, for example a slip as opposed to a trip, to which a person is susceptible in contrast to an any-cause fall. We consider specificity to be instrumental to the prescription of targeted, fall-specific prevention interventions.

Biomarker specificity addresses a key limitation of both currently acknowledged risk factors for falls and variables that have previously been suggested as potential biomarkers for falls. The limitation is that the risk factors and proposed biomarkers are associated with *all-cause* fall risk. Aside from slips, trips, and falls to the side, all cause falls also include those that occur while ascending or descending stairs, falls from bed, chairs, and ladders, falls due to carrying external loads, collisions, and using mobility assistance devices. In addition, there is also the ambiguous, if not necessary, fall type that include “other”, “non-classifiable,” and “unknown” that can often account for meaningful percentages of the total number retrospectively and prospectively reported falls (Talbot et al., 2005; Pai et al., 2014b).

Presently, our focus is on biomarkers of “direction-specific, preventable gait-related falls” by independent community-dwelling adults. By direction specific, we distinguish between trips that cause anteriorly-directed falls, slips that cause posteriorly-directed falls, and falls to the side. We operationally define a preventable gait-related fall as one that occurs by a community-dwelling adult while in the home or in the community during conditions in which the external and/or internal (prevailing physiological conditions) environments do not limit the initiation and completion of a compensatory-stepping response. To this last point, following a loss of balance that, unless corrected, would inevitably lead to a fall, the physical environment in which the loss of balance occurs can exert a substantial effect on the probability of performing a temporally and spatially sufficient stepping response to avoid a fall. If a particular environment shortens either the time available to perform a stepping response or decreases the physical space that would accommodate the stepping response, then the probability of successfully performing the stepping response is diminished. Similarly, a sudden change in blood pressure, onset of dizziness, or a change in vision could render a loss of balance unrecoverable. We offer five premises in support of our argument that discovery of biomarkers of “direction-specific, preventable gait-related falls” can increase the effectiveness of fall-prevention interventions.

### Premise 1

*From a practical perspective, some gait-related falls by older adults are preventable and some are not preventable*. An important characteristic of preventable gait-related fall is that neither the internal nor external environments preclude or limit the execution of a successful stepping response. Rather, it is the functional capacity of an individual to initiate and complete successful stepping response being exceeded by the requirements imposed by external and/or internal environments. Improved functional capacity is the focus of a fall-prevention intervention.

### Premise 2

*Different types of preventable gait-related falls by older adults may not be equally preventable*. This premise remains to be experimentally confirmed or refuted. However, for example, once initiated, avoiding a slip-related fall appears to be more challenging for older adults than avoiding trip-related fall. Following a laboratory-induced trip, 100% young adults were able to avoid a fall (Grabiner et al., 1993, 1996). Under similar conditions, following a laboratory-induced slip using a slippery surface mimicking ice, 74% of older adults were able to avoid a fall (Pavol et al., 2001; Grabiner et al., 2012). However, following a laboratory-induced slip on a slippery surface, 86% of young adults were able to avoid a fall, but only 14% of older adults were able to do so (Troy et al., 2008). Neural and biomechanical explanations for the apparent greater difficulty by older adults to avoid a slip-related fall compared to a trip-related fall have been proposed (Grabiner et al., 2014).

### Premise 3

*The potential for interventions to decrease the incidence of preventable gait-related falls by older adults may not be similar for different fall types*. This premise remains to be experimentally confirmed or refuted. The rationale for this premise follows logic similar to that for slip-specific and trip-specific falls noted in Premise 2. Aging-related degradation of the neuromuscular and musculoskeletal systems that includes, but is not limited to muscle strength, muscle power, flexibility, coordination, and response time, are normal and expected although there is substantial between-person variability. Collectively, these aging-related changes render the recovery solution for slip-related falls an increasingly challenging motor skill for older adults, in general, to perform, acquire, retain, and generalize/transfer the skill to different tasks having similar performance attributes (e.g., König et al., 2019). Therefore, the deleterious aging-related changes to the motor fitness may require an intervention approach requiring longer intervention time and/or larger exposure to slow or reverse.

### Premise 4

*Certain types of preventable gait-related falls may not be as prevalent as others*. *Most of the data regarding the prevalence of fall types has been acquired retrospectively and has the limitations that come with that type of reporting*. Nevertheless, slips and trips combined generally represent the largest reported proportion of gait-related falls by older adults. Prospectively-measured trip-related falls appear to occur more frequently than slip-related falls by community-dwelling adults (Berg et al., 1997; Hill et al., 1999; Decullier et al., 2010; Pai et al., 2014b; Crenshaw et al., 2017), by older adults in long-term care (Robinovitch et al., 2013), and older adults with intellectual disabilities (Smulders et al., 2013). Limited evidence suggests that gait-related falls that occur to the side may occur at rates that approach those of trips and slips (Stevens et al., 2014; Crenshaw et al., 2017). Similar to trip-related and slip-related falls, many falls that occur to the side may potentially be preventable by executing a temporally and spatially appropriate compensatory stepping response. Indeed, laterally-directed stepping responses have been shown to be improved by task-specific training, at least for young adults (Hurt et al., 2011).

### Premise 5

Preventable gait-related falls have stepping response solutions, which as motor skills, may be practiced, acquired, and retained. Our definition of a preventable gait-related fall specifies that the external and/or internal environments do not limit the initiation and completion of a stepping response.

In addition to the above premises, our focus on biomarkers of “direction-specific preventable gait-related falls” by independent community-dwelling adults reflects the growing magnitude of fall-related issues, the range of which spans from the individual older adults and their families to societal. In 1960, there were approximately 16.6 million adults 65 years of age and older in the United States (U.S. Bureau of the Census, 1996). In 2012 this number had more than doubled to about 43 million, is expected to double once again by 2050, and further increase to 98 million by 2060 (Colby and Ortman, 2014). In 2012 there were 3.2 million non-fatal falls by older adults reported in the US. Given that about 25% of older adults are estimated to fall annually (Bergen et al., 2016; Centers for Disease Control Prevention, 2018) the number of falls is under-reported. Of the reported non-fatal falls in 2012, 18% required hospitalization, 55% involved an emergency department visit, and 27% led to office-based/outpatient visits (Burns and Stevens, 2016). The estimated total medical-related cost of these non-fatal, reported falls in 2012 was $30.3 billion.

Based on the 2012 statistics above, and assuming that the percentage of serious fall-related injuries and/or costs of medical care for these injuries will not change appreciably, the costs for treating 6.5 million reported non-fatal falls by older adults will logically increase proportionately to the population by 49% to $45 billion. Overall, at the societal level, the costs to the government of healthcare are the focus of a great deal of political energy. That said, at the level of individual older adults and their families, the specter of fall-related morbidity including the loss of mobility and independence, mortality, and financial burden represent a source of everyday concern for many, if not most older adults.

Some fall-related injuries, such as hip fracture, head injury, and spine injury impose significant morbidity and post-injury mortality. For example, 20% of hip fracture survivors require long-term medical care. Notably, in the US, Medicare will not pay for long-term medical care and <15% of older adults in the US have long-term care insurance (Tajeu et al., 2014). Ignoring, for the moment, the expectation that the Medicare Hospital Insurance Trust Fund has been projected to become depleted by about 2030 (Centers for Medicare Medicaid Services, 2019), for many, if not most older adults, personal retirement savings is insufficient to bridge the gap between the total medical costs of a fall-related injury and that provided by Medicare coverage. Therefore, a reasonable potential solution, albeit partial, to this is to prevent the injurious fall from occurring in the first place.

The above arithmetic is compelling. If the expected incidence of falls by older adults cannot be decreased beyond that which is currently possible, then the growth of the population of older adults assures that the number of falls and the corresponding personal and societal problems will increase proportionately, at least through the end of this century. This, then, represents a growing gap that is important to narrow if possible. We propose that the gap may be narrowed to some extent by three means. The first is to increase the percentage of older adults who engage is exercise. The second is to increase the effectiveness of clinical assessment of fall risk. The third is to increase the effectiveness of fall-prevention interventions. All three can be addressed by focusing attention on falls that are demonstrably preventable.

## Currently used fall-risk assessments have limited utility

The effectiveness of clinical tests that are commonly used to assess fall risk is dependent upon the population for which the assessment is desired. When applied to independent community-dwelling older adults, who represent more than 95% of the entire population of older adults (Institute on Aging; Ortman et al., 2014), systematic reviews and meta-analyses have consistently reported that included studies were of low to moderate quality, the sensitivity of many presently used clinical fall risk assessment methods is low, sometimes unacceptably low, and, consequently did not warrant the authors recommendations (Scott et al., 2007; Gates et al., 2008; Muir et al., 2010; Beauchet et al., 2011; Rydwik et al., 2011; Schoene et al., 2013; Omaña et al., 2021).

## Exercise-based Interventions to Reduce fall risk are Effective but participation rates by older adults are low

Fall prevention interventions are effective at reducing falls and exercise may be the most effective fall prevention intervention. Exercise decreases fall incidence by about 23% and decreases fall rate between 17 and 29% (Sherrington et al., 2019). Of the falls that occur and are reported, exercise decreases injurious falls by about 50% (Tricco et al., 2017). However, the participation rate by older adults in exercise is low. Based on the US guidelines for leisure time activity, fewer than 30% of adults older than 75 years of age meet the aerobic activity guidelines (Clarke et al., 2017). Fewer than 9% of adults older than 75 years of age meet the guidelines for aerobic plus muscle strengthening activities (Clarke et al., 2017). Thus, we suggest that exercise, the most robust fall-prevention intervention, likely falls quite short of its potential. *However, increased participation rate in exercise programs by older adults, in addition to increased adherence to the programs, in conjunction with increased effectiveness, that is the specificity of exercise to target fall prevention, represent achievable targets that could effectively decrease the incidence of falls*.

## The case for potential biomechanical biomarkers for falls and a path forward

Establishing biomechanical biomarkers of preventable, gait-related, and cause-specific falls is achievable. The extent to which a biomarker for falls qualifies as causal can be evaluated using the Bradford Hill criteria (Hill, 1965; Aronson, 2005). These criteria are possibly the most frequently cited framework for inferring causality (Fedak et al., 2015). The nine criteria are strength of association, consistency, specificity, temporality, biological gradient (dose-response), plausibility, coherence, experiment, and analogy. Accumulating evidence convincingly demonstrates that sagittal plane trunk kinematics of older adults following trip-specific treadmill-delivered disturbances and following laboratory-induced trips meet many of the Hill criteria.

The first criterion, strength of association, is met by sagittal plane trunk kinematics following a laboratory-induced trip. Sagittal plane trunk kinematics are positively, strongly, statistically significantly, and repeatably related to the outcome (fall or recovery) of a laboratory-induced trip. The positive association between trunk flexion angle, measured at the recovery step completion (the instant at which the foot of the recovery step limb contacts the ground) and trip-related falls was demonstrated when young adults and older adults were subjected to laboratory-induced trips (Grabiner et al., 1993, 1996, 2012, 2014; Pavol et al., 2001).

Following a laboratory-induced trip the difference between the trunk flexion angle at recovery step completion of older adults who did not fall and that of those who did fall was significant (Pavol et al., 2001; Grabiner et al., 2012). The 95% confidence intervals for trunk flexion angle at recovery step completion for those who fell and for those who did not fall were (37.3–41.3°) and (20.4–26.4°), respectively. Further, the 95% confidence interval for trunk flexion angle at recovery step completion of the young adults, who did not fall, overlapped with that of the older adults who did not fall (23.3–31.5°). Collectively, these results are also consistent with the fifth Hill criterion of biological gradient, or dose-response. This is similarly so for the trunk velocity at recovery step completion. The 95% confidence intervals for the older adults who did not fall and that of those who did fall were −35 to −14°/s (the negative sign indicates trunk extension velocity) and 36–72°/s (the positive values indicate trunk flexion velocity), respectively (Pavol et al., 2001; Grabiner et al., 2014). The extension velocity of the trunk at recovery step completion means that these older adults were able to both arrest and reverse the trunk velocity prior to contact of the recovery foot with the ground.

The second Hill criterion, consistency, refers to the extent that the association has been reported by different laboratories and/or for different populations. In addition to healthy, community-dwelling older adults, similar relationships have been reported between falls following trip-specific treadmill-delivered perturbations and trunk kinematics for people with unilateral, bilateral, below-knee, and above-knee lower extremity amputation (Kaufman et al., 2014), for women with knee joint osteoarthritis (Pater et al., 2016; Foucher et al., 2020), and post-stroke patients (Honeycutt et al., 2016). However, it warrants noting that recently, a protocol consisting of both trip-specific and-slip specific training was reported to not have had an effect on trunk flexion angle by older adults at recovery step completion following a laboratory-induced trip (Allin et al., 2020).

The third criterion, specificity, restricts the association to an explicit outcome. Trip-specific training appears to decrease prospectively measured falls by middle age and older women following laboratory-induced trips during overground walking (Grabiner et al., 2012). Subsequent to that work, trip-specific training appears to have decreased prospectively measured trip-specific fall rate over a 12 month period by middle age and older women by about 50% (Rosenblatt et al., 2013). Notably, the trip-specific training was not associated with a change in the rate of falls due to causes other than trips. To date, the results of Grabiner et al. (2012) and Rosenblatt et al. (2013) have not been subjected to replication studies. However, in another study, young and older adults were subjected to both forward and backward platform-delivered perturbations. The stepping responses of older adults were improved in both directions. However, the stepping responses of young adults improved only in the forward direction. Importantly, in the present context, neither the older nor younger adults improved following laterally-directed perturbations (Dijkstra et al., 2015). Recently, trip-specific training was reported as superior to Tai Chi training with regard to reducing trunk flexion following treadmill-delivered trip-specific disturbances (Avils et al., 2019). This may be the first published, direct comparison of the efficacy of trip-specific training to Tai Chi, which has become a standard fall-prevention intervention (Stevens and Burns, 2015). Collectively, the published literature suggests that the effects of trip-specific perturbation training are, indeed, specific to trip-related falls.

The fourth criterion, temporality, specifies that the effect cannot occur before the cause. Trunk flexion velocity at recovery step completion, discussed in a previous section on the first criterion, fulfills this criterion. In the present context, trunk flexion velocity, in particular, is viewed as a biomechanical cause and the fall is the effect. Similar to trunk angle, the trunk angular velocity is measured at recovery step completion. This means that those older adults who did not fall were able to *arrest* and *reverse* the trip-induced trunk flexion velocity prior to recovery step completion. In contrast, the trunk velocity of those who fell was in the direction of flexion. A similar finding was reported from an experiment in which laboratory-induced trips were delivered to middle age and older women who had participated in a trip-specific perturbation protocol or who had served in the control group (Grabiner et al., 2012). This also addresses the criterion of consistency.

The fifth criterion is biological gradient, or dose-response. This criterion appears to be met by the extent to which increased trunk flexion angle and direction of trunk velocity are related to the probability of a fall. Following a laboratory-induced trip, at recovery step completion, the trunk flexion angle of the women who fell was 37 ± 5° and the trunk velocity was 40 ± 41°/s (flexion). In contrast, the trunk flexion angle of the women who did not fall was 22 ± 13° and the trunk velocity was −13 ± 44°/s (extension). The between-group differences for trunk flexion angle and trunk velocity were significant. Interestingly, the recovery step length of women who fell was significantly shorter than that of women who did not fall (28.4 ± 19.3% body height vs. 58.2 ± 16.9% body height, respectively. This result was consistent with a suggestion that, in addition to decreasing dynamic stability, increased trunk flexion following a trip would interfere with performance of the recovery step through the action of antagonistic biarticular muscles (Grabiner et al., 1993).

The remaining four Hill criteria include plausibility, coherence, experiment, and analogy. The biomechanical underpinnings of the causal biomechanical roles of trunk flexion angle and trunk angular velocity are plausible, given that that the head, arms, and trunk represent about 50% of the total body mass. The demonstrable and expected age-related decrease in trunk muscle strength and muscle power would reasonably be predicted to require more time and/or distance, i.e., range of motion, to arrest and reverse the motion of this mass caused by the trip. Coherence refers to the extent to which the association is consistent with generally available knowledge. Hill's criterion of experiment relates to the evidentiary strength for claims of causality of experimentally derived results when supported by the established association. Lastly, using the criterion of analogy, Hill suggested that, in some cases, having established a variable as causally related to an outcome may lower the standard of evidence for subsequent variables if they are, in some way, related to those previously established.

In the aggregate, the established relationship between trunk kinematics following trip-specific perturbations and the subsequent success or failure of the recovery efforts appear to meet many of the requisite cause-effect characteristics defined by Hill (1965). In addition, trunk kinematics following a trip-specific perturbation appear to meet many of the general requirements for biomarkers. The kinematic variables may be accurately measured, they are clinically feasible and are non-invasive. Furthermore, they are clinically modifiable and direction specific. However, there are criticisms of perturbation-based fall prevention. A frequently raised criticism is the scalability and cost-effectiveness that reflect the cost and availability of the required instrumentation. This is a legitimate concern although one could argue that the cost-benefit is reasonable, especially if the issue of “moving the needle” with respect to fall prevention is of sufficient importance to reconsider the resources to be invested in the necessary infrastructure. Further, the previously described limitations of existing clinical tests of fall risk and the very low rates of exercise participation by older adults must be recognized as elements in the equation. Another frequently raised criticism related to perturbation-based fall prevention interventions has been directed at one of its strengths, that is, its specificity. Trip-specific perturbation training appears to decrease prospectively measured falls following laboratory-induced trips (Grabiner et al., 2014) and prospectively measured trip-related fall rate in the community (Rosenblatt et al., 2013). It warrants mention that the study design of Rosenblatt et al. (2013) was designed to be powered, a priori, to detect a 50% reduction in all-cause falls and not trip-specific fall rate. Nevertheless, trip-specific perturbation training did not appear to have an effect on other types of falls. To date, the experiment of Rosenblatt et al. (2013) has not had the benefit of being repeated. Nevertheless, the implication is that trip-specific protocols may have to be conducted separately from slip-specific protocols and protocols directed at reducing falls to the side. This, of course increases the time demands of the protocols. However, prior to crossing that bridge it seems reasonable to first demonstrate if perturbation-based interventions are actually effective in reducing the incidence of these type of falls in the community.

It is abundantly clear from published data that trunk kinematics improve as a result of trip-specific perturbation training and the published literature points to this improvement being causally associated with decreased probability of a trip-specific fall. What remains to be done, from our point of view, is to systematically determine if the trunk biomarkers measured on an individual who has not participated in an intervention, general, or trip-specific, can prospectively predict trip-specific falls by that individual. This will prove challenging. First, exposure to even a single trip-specific perturbation is sufficient to induce a significant improvement in the performance of the recovery of a second perturbation (Owings et al., 2001). Secondly, prior knowledge of an imminent perturbation, such as that disclosed during the informed consent, is sufficient to induce a significant improvement in the performance of the recovery, at least for young adults (Oludare et al., 2018). Challenging as it may be, the clinical value of having a subject-specific baseline record, in the form of biomarkers for trip-specific falls, would allow quantitatively informed opinions regarding the effectiveness of an intervention, general or trip-specific, to reduce trip-specific fall risk. In addition, such a baseline and follow-up records could contribute to a clinical decision regarding when, or if, the maximum effect of an intervention had been achieved for an individual.

We previously described how our work related to trip-specific falls followed the model originally proposed by Mercy et al. (1993). In light of the reasonably positive outcomes of having done so, we propose that, moving forward, this same approach may be useful to consider and apply to other types of potentially preventable gait related falls, particularly slip-related falls, and falls that occur to the side. Both of these types of falls share a common biomechanical characteristic critical to the recovery solution(s). The common characteristic is that successfully avoiding a fall is reliant, to a large extent, on the ability to execute an appropriate stepping response within the available time and space.

### Slip-Related Falls

There is ample evidence to support the claim of efficacy of slip-specific perturbation training. That is, slip-specific perturbation training has repeatably been reported to decrease falls following platform-induced slips in the laboratory by older adults (e.g., Pai et al., 2010, 2014a; Lee et al., 2018; Okubo et al., 2019; Wang et al., 2019; Allin et al., 2020).

Thus far, though, the effectiveness of slip-specific perturbation training to reduce slip-related falls in the community is not known. To our knowledge there may be only one published study reporting the effects of slip-specific training on prospectively measured slip-specific falls (Pai et al., 2014b). In this study, 67 community-dwelling older adults were subjected to a series of 24 slip-specific perturbations using a sliding platform. A control group of 75 community-dwelling older adults were subjected to a single slip-specific perturbation. Subsequently, all-cause falls, of which there were five types, were prospectively assessed for 12 months. The fall types were slips, trips, those that were caused by ADL and transfers, those that were caused by external hazards, and those for which the causes were categorized as “others/unknown.” The between-group difference for all-cause falls, i.e., inclusive of all fall types, achieved significance. However, the study was not designed to directly compare between-group difference for slip-related falls (20 vs. 16.7% of the total number of falls for the trained and control groups, respectively, for the on-treatment analysis; 28.6 and 15.4% of the total number of falls for the trained and control groups, respectively, for the intention-to-treat analysis). It is possible that the significant main effect of the perturbation training may have been influenced by the number of falls that were categorized as “other” or “unknown.” In this category of falls, the percentages of the total number of falls for the control group were more than two and six times that of the training group for the on-treatment analysis and intention-to-treat analysis, respectively. Nevertheless, based only on the results of this single experiment which, similar to the study of Rosenblatt et al. (2013), has not had the benefit of being repeated, a conclusion related to the effectiveness of slip-specific perturbation training on the incidence of slip-related falls in the community by older adults, is premature. That said, there is a trial currently being conducted, but for which the results do not yet appear to have been disseminated, that is focused on the effects of slip- and trip-specific perturbation training on the prospectively measured slip- and trip-specific falls (Rieger et al., 2020).

### Falls to the Side

At present and to our knowledge, there is no published research related to the use of perturbation training to decrease the incidence *laterally-directed* falls either in the laboratory or in the community. Falls to the side, which may account for up to 33% of falls by older adults (Crenshaw et al., 2017), are a particular concern because of their association with hip fracture. Hip fracture is the cause of a high level of morbidity, mortality, debility, and destitution in the population of older adults (Tajeu et al., 2014). However, in the light of the framework for evaluating causality, it is notable that the ability to perform laterally-directed stepping responses following waist-pull perturbations has been associated with prospectively measured all-cause falls, by older adults (Hilliard et al., 2008; Mille et al., 2013; Rogers et al., 2021). For young adults, laterally-directed stepping following treadmill-delivered disturbances is improved by practice (e.g., Hurt et al., 2011). In another experiment, young adults walking on a treadmill were subjected to direction-specific waist-pull perturbations. The quality of the stepping responses was measured as the margin of stability (Hof et al., 2005) and improved in the anterior-posterior direction, but not the medial-lateral direction (Martelli et al., 2017). In the aggregate, although the ability to perform stepping responses following laterally-directed perturbations appear to be clinically modifiable, at present there does not appear to be established risk factors or biomechanical biomarkers for laterally-directed falls.

The rate of growth of the population of older adults, for which a slowing has not been predicted, has outpaced the ability of currently used clinical interventions and general exercise participation by older adults to reduce the incidence of falls by older adults. The ensuant health and health care issues, which cannot be easily separated from political and economic issues, will likely continue to grow in parallel. We have tried to make the case for systematically establishing biomarkers for falls by older adults. We have proposed that a family of biomechanical biomarkers for direction-specific and gait-related falls can increase the effectiveness of fall prevention interventions and, potentially, serve as indices for the extent to which an intervention has achieved or is nearing its maximum effect for a specific individual. In this case, the maximum effect is with regard to preventable, direction-specific, gait-related falls by older adults. Presently, perturbation training directed toward decreasing the incidence of trip-related falls by older adults has the largest body of supportive evidence supporting both its efficacy and effectiveness. The efficacy of perturbation training to reduce slip-related falls by older adults appears to have been confirmed although its effectiveness of to reduce slip-related falls by older adults is an open and important question. Both the efficacy and effectiveness of perturbation training to reduce falls to the side falls by older adults, understudied and reported, represents an important research opportunity. In light of the exponential nature of the growth of both the population of older adults and the incidence of falls, particularly injurious falls, the present seems to be a perfect time to at least consider the possibility that we can do better at refining and implementing the state-of-the art with regard to fall prevention.

A key challenge to the design of prospective experiments that will be necessary to convincingly confirm or refute efficacy and effectiveness of perturbation training to reduce direction-specific falls by older adults to is the estimated sample size. Sample sizes will necessarily be much larger to ensure *a priori* statistical power to detect between group-differences in the incidence of falls due to direction-specific types of falls such as trips, slips and falls to the side compared to all-cause falls (Karamanidis et al., 2020). This translates to higher costs and more time to conducting such studies. We support the recommendation for a solution involving large, collaborative efforts (Karamanidis et al., 2020). Such an collaborative team approach has the potential to link international research and clinical facilities, scientists and clinicians, thereby serving as a force multiplier for shared and increasingly important interests.

## Author Contributions

Both authors contributed equally to the drafting and revising of this manuscript.

## Conflict of Interest

The authors declare that the research was conducted in the absence of any commercial or financial relationships that could be construed as a potential conflict of interest.

## Publisher's Note

All claims expressed in this article are solely those of the authors and do not necessarily represent those of their affiliated organizations, or those of the publisher, the editors and the reviewers. Any product that may be evaluated in this article, or claim that may be made by its manufacturer, is not guaranteed or endorsed by the publisher.
